# *Toxoplasma-*Induced Hypermigration of Primary Cortical Microglia Implicates GABAergic Signaling

**DOI:** 10.3389/fcimb.2019.00073

**Published:** 2019-03-20

**Authors:** Amol K. Bhandage, Sachie Kanatani, Antonio Barragan

**Affiliations:** Department of Molecular Biosciences, The Wenner-Gren Institute, Stockholm University, Stockholm, Sweden

**Keywords:** apicomplexa, central nervous system, glia, leukocyte migration, neurotransmission, GABA receptor

## Abstract

*Toxoplasma gondii* is a widespread obligate intracellular parasite that causes chronic infection and life-threatening acute infection in the central nervous system. Previous work identified *Toxoplasma*-infected microglia and astrocytes during reactivated infections in mice, indicating an implication of glial cells in acute toxoplasmic encephalitis. However, the mechanisms leading to the spread of *Toxoplasma* in the brain parenchyma remain unknown. Here, we report that, shortly after invasion by *T. gondii* tachyzoites, parasitized microglia, but not parasitized astrocytes, undergo rapid morphological changes and exhibit dramatically enhanced migration in 2-dimensional and 3-dimensional matrix confinements. Interestingly, primary microglia secreted the neurotransmitter γ-aminobutyric acid (GABA) in the supernatant as a consequence of *T. gondii* infection but not upon stimulation with LPS or heat-inactivated *T. gondii*. Further, microglia transcriptionally expressed components of the GABAergic machinery, including GABA-A receptor subunits, regulatory molecules and voltage-dependent calcium channels (VDCCs). Further, their transcriptional expression was modulated by challenge with *T. gondii*. Transcriptional analysis indicated that GABA was synthesized via both, the conventional pathway (glutamate decarboxylases GAD65 and GAD67) and a more recently characterized alternative pathway (aldehyde dehydrogenases ALDH2 and ALDH1a1). Pharmacological inhibitors targeting GABA synthesis, GABA-A receptors, GABA-A regulators and VDCC signaling inhibited *Toxoplasma*-induced hypermotility of microglia. Altogether, we show that primary microglia express a GABAergic machinery and that *T. gondii* induces hypermigration of microglia in a GABA-dependent fashion. We hypothesize that migratory activation of parasitized microglia by *Toxoplasma* may promote parasite dissemination in the brain parenchyma.

## Introduction

*Toxoplasma gondii* is a globally widespread parasite that infects virtually all warm-blooded organisms, including humans and rodents (Joynson and Wreghitt, 2001). Chronic carriage of *T. gondii* without major symptomatology is common. However, systemic dissemination of *T. gondii* can cause life-threatening infection that manifests as *Toxoplasma* encephalitis in immune-compromised patients (Joynson and Wreghitt, 2001). *T. gondii* is obligate intracellular and the tachyzoite stage actively invades and replicates within nucleated cells in the host (Frénal et al., 2017). Tachyzoites subvert the migratory properties of leukocytes, e.g., dendritic cells (DCs) (Lambert et al., 2006), and use shuttle leukocytes (Trojan horse) to rapidly reach the blood circulation and the central nervous system (CNS) (Courret et al., 2006; Lambert et al., 2006, 2009). Fast-replicating tachyzoite stages can infect microglia, astrocytes, and neurons *in vitro* (Lüder et al., 1999; Scheidegger et al., 2005).

Microglia are the principal resident immune cell in the CNS and originate from primitive hematopoietic precursors outside the CNS, in the embryonic yolk sac (Ginhoux et al., 2010). Microglia rapidly respond to tissue injury and inflammation (Kreutzberg, 1996; Nimmerjahn et al., 2005). They are likely an important source of IFN-γ and interact with CD4^+^ and CD8^+^ lymphocytes, thus contributing to acquired immunity during toxoplasmic encephalitis (Suzuki et al., 2005). Astrocytes also play important roles in resistance to *T. gondii* during chronic infection (Wilson and Hunter, 2004). Thus, multiple functions have been attributed to glia cells during *Toxoplasma* infection in murine models, including cytokine production and phagocytosis (Strack et al., 2002; Wilson and Hunter, 2004; Suzuki et al., 2005; Carruthers and Suzuki, 2007). Microglial nodules in the CNS have also been described during *Toxoplasma* infection in humans (Nebuloni et al., 2000). Yet, the mechanisms for parasite dissemination locally in the CNS parenchyma, that is associated with life-threatening encephalitis, remain unknown.

Γ-aminobutyric acid (GABA), the main inhibitory neurotransmitter in the vertebrate brain, has also been attributed motogenic functions outside the CNS including cell migration and metastasis (Azuma et al., 2003; Wheeler et al., 2011). Along these lines, *Toxoplasma* induces hypermigration of infected DCs through GABAergic signaling (Fuks et al., 2012; Kanatani et al., 2017). GABA-A receptors (GABA-A R) are ionotropic chloride channels composed from pentameric combinations of 19 different subunits (Olsen and Sieghart, 2008) and whose functions are regulated by cation-chloride co-transporters (CCCs) (Kahle et al., 2008). GABA is shuttled in and out of cells via GABA transporters (GAT) (Höglund et al., 2005) and GABAergic cells synthesize GABA via glutamate decarboxylases (GAD65/67) and can metabolize it by GABA-transaminase (GABA-T) (Soghomonian and Martin, 1998). GABA can also be synthesized from putrescine (Seiler et al., 1973) and more recent work has characterized an alternative GABA synthesis pathway via monoamine oxidase B (MAOB) and aldehyde dehydrogenases (ALDH2 and ALDH1a1) in neurons and astrocytes (Yoon et al., 2014; Kim et al., 2015). Thus, GABAergic signaling has been extensively studied in neurons and astrocytes (Lee et al., 2011; Kilb, 2012) but remains chiefly unexplored in microglia (Barragan et al., 2015; Bhandage and Barragan, 2019).

Here, we report that migratory activation of *Toxoplasma*-infected microglia occurs via GABAergic signaling. We discuss the possible implications of GABA secretion and the migratory activation of microglia in the pathogenesis of *Toxoplasma* encephalitis.

## Materials and Methods

### Parasites and Cell Line

*Toxoplasma gondii* lines used include GFP-expressing PTGluc (type II, cloned from ME49/PTG-GFPS65T) and RFP-expressing PRU-RFP (type II). Tachyzoites were maintained by serial 2-day passaging in human foreskin fibroblast (HFF-1 SCRC-1041, American Type Culture Collection) monolayers cultured in Dulbecco‘s modified Eagle’s medium (DMEM; Thermofisher scientific) with 10% fetal bovine serum (FBS; Sigma), gentamicin (20 μg/ml; Gibco), glutamine (2 mM; Gibco), and HEPES (0.01 M; Gibco), referred to as complete medium (CM). The murine microglia cell line BV2 (American Type Culture Collection) was cultured in CM.

### Primary Glia Cells and Microglia

Primary glia cell cultures were generated as follows. One- to three-day-old pups from C57BL/6 mice were euthanized. The brains were dissected and cortices collected. Cortices were further washed in ice-cold Ca^2+^- and Mg^2+^ free Hanks' buffered salt solution (HBSS; Gibco), minced, and resuspended in ice-cold HBSS. After being washed, tissues were incubated for 15 min in HBSS containing 0.1% trypsin and resuspended in astrocyte medium containing DMEM F-12 (Gibco), 10% FBS, 1% G5 supplement (Gibco), and gentamicin (20 μg/ml; Gibco). Medium was changed every 2–3 days. Microglia were harvested as described previously (Dellacasa-Lindberg et al., 2011). Briefly, confluent astrocyte monolayers derived from primary glia cultures were sub-cultivated in microglia medium containing DMEM F-12, 10% FBS, glutamine (2 mM; Gibco), and gentamicin (10 μg/ml). Microglial cells were harvested from confluent astrocyte monolayers after 7 days by tapping the side of the culture flasks, removing loosely adherent microglia from astrocyte monolayers. Microglia purity was assessed by transcriptional analysis of a marker panel (Figure S1 and Table S1).

### Reagents

Lipopolysaccharide (LPS), 4-Diethylaminobenzaldehyde (DEAB; aldehyde dehydrogenase inhibitor), selegiline (MAO-B inhibitor), nifedipine (L-type VDCC inhibitor, all from Sigma-Aldrich), L-allylglycine (L-AG; GAD inhibitor), picrotoxin (GABA-A receptor inhibitor), bumetanide (NKCC1 inhibitor), benidipine (broad VDCC inhibitor, all from Tocris Bioscience), and 1-(3-Chlorophenethyl)-3-cyclopentylpyrimidine-2,4,6-(1H,3H,5H)-trione (CPCPT, CaV 1.3 inhibitor, Merck Millipore) were used at the indicated concentrations. Heat inactivation of *T. gondii* tachyzoites was performed at 56°C for 30 min. Supernatants were collected from microglia incubated with freshly egressed *T. gondii* tachyzoites (MOI 1, 24 h) and added at a final concentration of 1:1.

### Motility Assays

Motility and velocity analyses were performed as previously described (Weidner et al., 2013). Briefly, microglia were challenged with freshly egressed tachyzoites or treated with antagonists. Cells were seeded on 96-well plates or matrigel-coated (100 μg/cm^2^, Corning) labtech chambers (Thermofisher). The cells were imaged every min for 60 min (Zeiss Observer Z.1). Motility patterns for 50–60 cells per experimental group were compiled using ImageJ (image stabilizer software and manual tracking plugins). Transmigration assays with astrocytes were performed in transwell filters (8 μm pore size, BD Falcon), as previously described (Fuks et al., 2012). Pharmacological inhibitors were added at concentrations that non-significantly impacted on cell morphology, baseline motility of unchallenged cells, and on parasite infection rates (Fuks et al., 2012).

### 3D Migration Assay

The assay was performed as previously described (Kanatani et al., 2015). Briefly, a collagen layer was prepared using bovine collagen I (0.75 mg/ml, GIBCO) in a 96-well plate (80 μl/well). Microglia were challenged with freshly egressed *T. gondii* tachyzoites (MOI 3) in CM for 4 h. The cell suspension (5 × 10^4^ cells) was applied to the collagen layer and incubated for 18 h at 37°C and 5% CO_2_. Gels were fixed and DAPI-stained. Image stacks were generated (200 optical sections) by confocal microscopy (LSM 780, Zeiss) and migrated distances by cells were analyzed using Imaris x64 v.8.1.1 software (Bitplane AG, Zurich, Switzerland).

### Immunocytochemistry

Host cells were cultured on glass coverslips. Fixation was performed with 4% PFA in PBS for 15–20 min at RT. The cells were permeabilized using 0.5% Triton X-100 in PBS. To visualize host cell F-actin and podosomes, cells were stained with Alexa Fluor 488- or 594- or 647-conjugated phalloidin (Invitrogen). To probe GABA-A subunits, cells were incubated with rabbit anti-GABA-A R α3 polyclonal antibody (Alomone labs), rabbit anti-GABA-A R α5 polyclonal antibody (Synaptic system), and mouse anti-GABA-A R β3 monoclonal antibody (NeuroMab) ON at 4°C. Following staining with Alexa Fluor 488-conjugated secondary antibodies (Invitrogen) and DAPI, coverslips were mounted and imaged by confocal microscopy (LSM 780 or LSM 800, Zeiss).

### Scoring of Cell Morphology

Phalloidin-stained microglia were monitored by epifluorescence microscopy (Leica DMRB). For each preparation, 20–30 randomly chosen fields of view were assessed and an average of 100 cells were counted per condition. The microglia were scored based on morphological criteria, as previously described for DCs (Weidner et al., 2013).

- Cell shape—elongated vs. rounded
- Podosome structures—present vs. absent

### Real-Time Quantitative PCR

Total RNAs were extracted using Direct-zol miniprep RNA kits with TRIzol reagent (Zymo Research). First-strand cDNA was synthesized using Superscript III or IV Reverse Transcriptase (Invitrogen) using a standard protocol provided by manufacturer. Real-time quantitative PCR (qPCR) was performed in Rotor-Gene 6000 (Corbett Research) or QuantStudio 5 384 Optical well plate system (Applied Biosystem) in a standard 10 μl with the 2X SYBR FAST qPCR Master Mix (KAPA Biosystems) and gene specific primers using a standard amplification protocol followed by melt curve analysis. The gene specific DNA primer pairs were designed to cover all the transcripts available currently using GETprime or NCBI primer blast tool, ordered from Sigma-Aldrich and validated on whole brain homogenates (Table S2). The criteria for positive detection of a signal were presence of single peak at specific temperature in the melt curve and presence of a single band in agarose gel electrophoresis. The relative expression levels (2^−Δ*Ct*^) were calculated for each target relative to a normalization factor -geometric mean of reference genes, glyceraldehyde 3-phosphate dehydrogenase (GAPDH), and Actin-β or TATA-binding protein (TBP) and importin 8 (IPO8).

### GABA Enzyme-Linked Immunosorbent Assays (ELISA)

ELISA (Labor Diagnostica Nord, Nordhorn, Germany) was performed as previously described (Fuks et al., 2012). Briefly, microglia were plated at a density of 5 × 10^5^ cells per well and incubated for 24 h in presence of *T. gondii* tachyzoites or reagents, as indicated. GABA concentrations in supernatants quantified at a wavelength of 450 nm (VMax® Kinetic ELISA Microplate Reader, Molecular Devices).

### Statistical Analysis

Statistical analyses were performed using GraphPad Prism 7.0 (La Jolla, CA, USA) and R Stats Package version 3.0.2 (R Foundation for Statistical Computing, Vienna, Austria). The significance level was set to *p* < 0.05.

## Results

### Primary Cortical Microglia Exhibit Morphological Changes and Enhanced Migration Upon Infection With *T. gondii*

**Figure 1:**
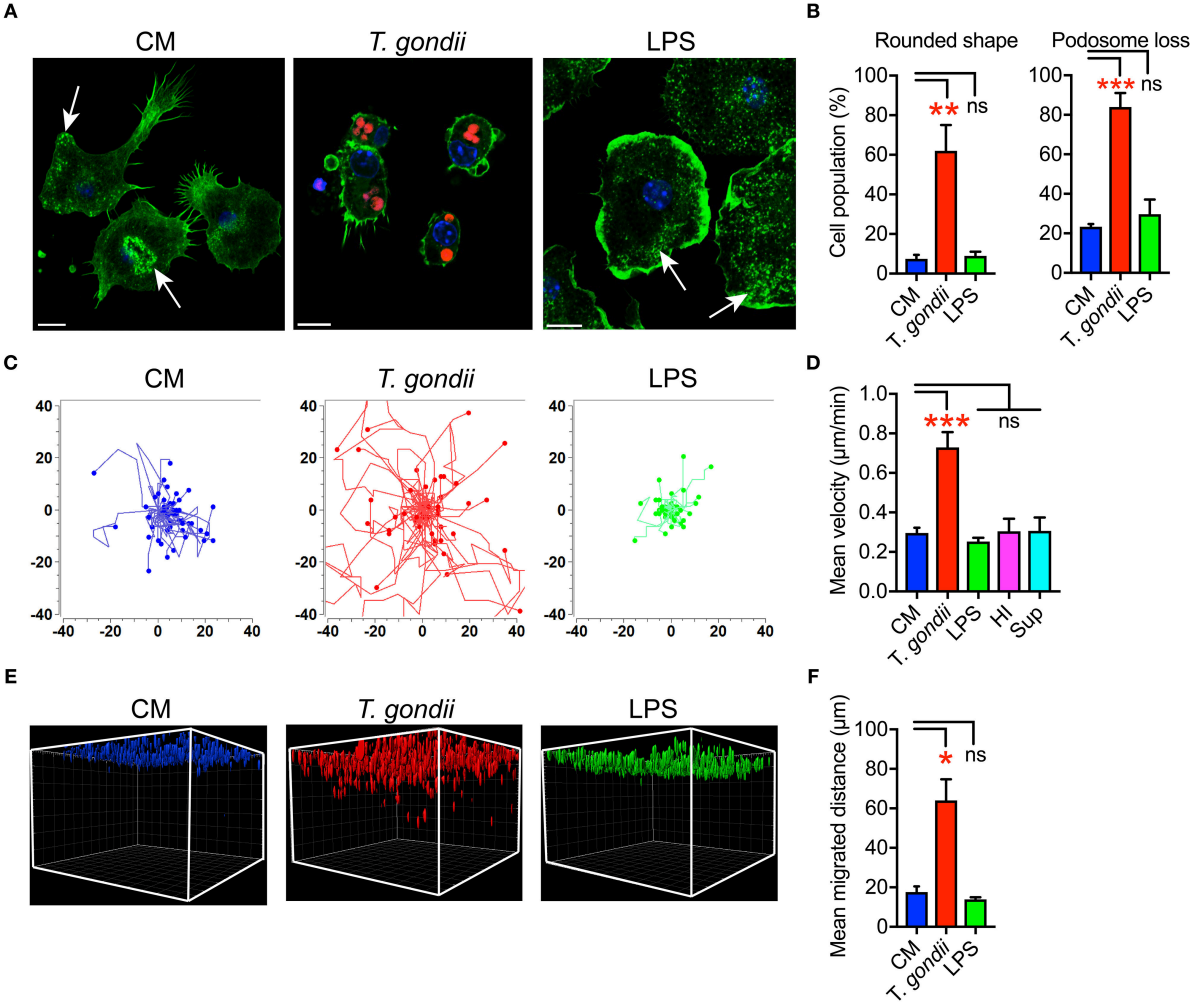
Morphological changes and hypermigration of *Toxoplasma-*challenged primary cortical microglia. **(A)** Representative micrographs of primary microglia stained with Alexa Fluor 488 Phalloidin to detect F-actin, as indicated under Materials and Methods. Primary murine microglia were incubated with freshly egressed RFP-expressing *T. gondii* tachyzoites (PRU-RFP, MOI 3), treated with LPS (100 ng/ml) or maintained in complete medium (CM), for 4 h. Arrows indicate F-actin condensation in podosome structures. Scale bar = 10 μm. **(B)** Percentage of cells that exhibited rounded cell shape and podosome loss, respectively, related to the total cell population analyzed for each condition from a total of 50–100 cells from 3 independent experiments. **(C)** Representative motility plots of microglia incubated with PTG tachyzoites for 4 h, complete medium (CM) and with LPS (100 ng/ml). **(D)** Mean velocities of microglia incubated with complete medium CM), PTG tachyzoites, heat-inactivated tachyzoites (HI), supernatant from *T. gondii-*infected microglia (Sup) are analyzed from 4 independent experiments. **(E)** Migration of microglia in matrix as indicated under Materials and Methods. One representative 3D reconstruction assembly of z-stacks is shown. Colored structures indicate the localization of individual microglia (DAPI) at indicated conditions. **(F)** Mean migrated distances by microglia under same condition as in **E**. Data represent compiled analysis of 500 randomly chosen cells from 2 independent experiments in duplicate. For **(B,D,F)**, bar graphs represent mean + SEM. Statistical significance was tested by One-Way ANOVA with Dunnett's *post-hoc* test. ns *p* ≥ 0.05, ^*^p < 0.05, ^**^p < 0.01, ^***^p < 0.001.

We previously described that *Toxoplasma*-infected DCs undergo rapid morphological changes and exhibit hypermotility in absence of chemotactic cues (Weidner et al., 2013). Similarly, infected microglia exhibited enhanced transmigration *in vitro* (Dellacasa-Lindberg et al., 2011). To further determine the cellular processes underlying this migratory activation, we characterized morphological changes in microglia upon challenge with *T. gondii* tachyzoites by staining F-actin filaments (Figure 1A). *Toxoplasma*-infected microglia were consistently characterized by loss of actin-rich cytoskeletal podosomes and a rounded morphology (Figures 1A,B). These observations were confirmed in the microglia cell line BV2 (Figure S2A). Next, a motility analysis was performed to address a possible link between the infection-associated morphological changes and the previously described enhanced transmigration of *Toxoplasma*-infected microglia (Dellacasa-Lindberg et al., 2011). Infected primary microglia and BV2 cells migrated significantly longer distances at higher velocities compared with unchallenged microglia or microglia challenged with LPS, heat-inactivated *T. gondii* or supernatant from infected microglia (Figures 1C,D and Figures S2B,C). This indicated that the migratory activation was linked to the presence of live intracellular parasites. In sharp contrast, infected primary astrocytes exhibited undistinguishable morphological changes compared to uninfected astrocytes (Figure S3A) and non-significant changes in motility (Figures S3B,C). Further, we analyzed the migratory activation of *Toxoplasma*-challenged microglia in a collagen matrix (Kanatani et al., 2015). In this 3D setting, infected microglia and BV2 cells migrated significantly longer distances compared with untreated or LPS-treated microglia (Figures 1E,F and Figure S2D). Altogether, we conclude that, upon infection with *T. gondii*, microglia undergo morphological changes and exhibit enhanced migration in 2D and 3D confinements.

### Expression of GABAergic and VDCC Signaling Components by Primary Cortical Microglia

**Figure 2:**
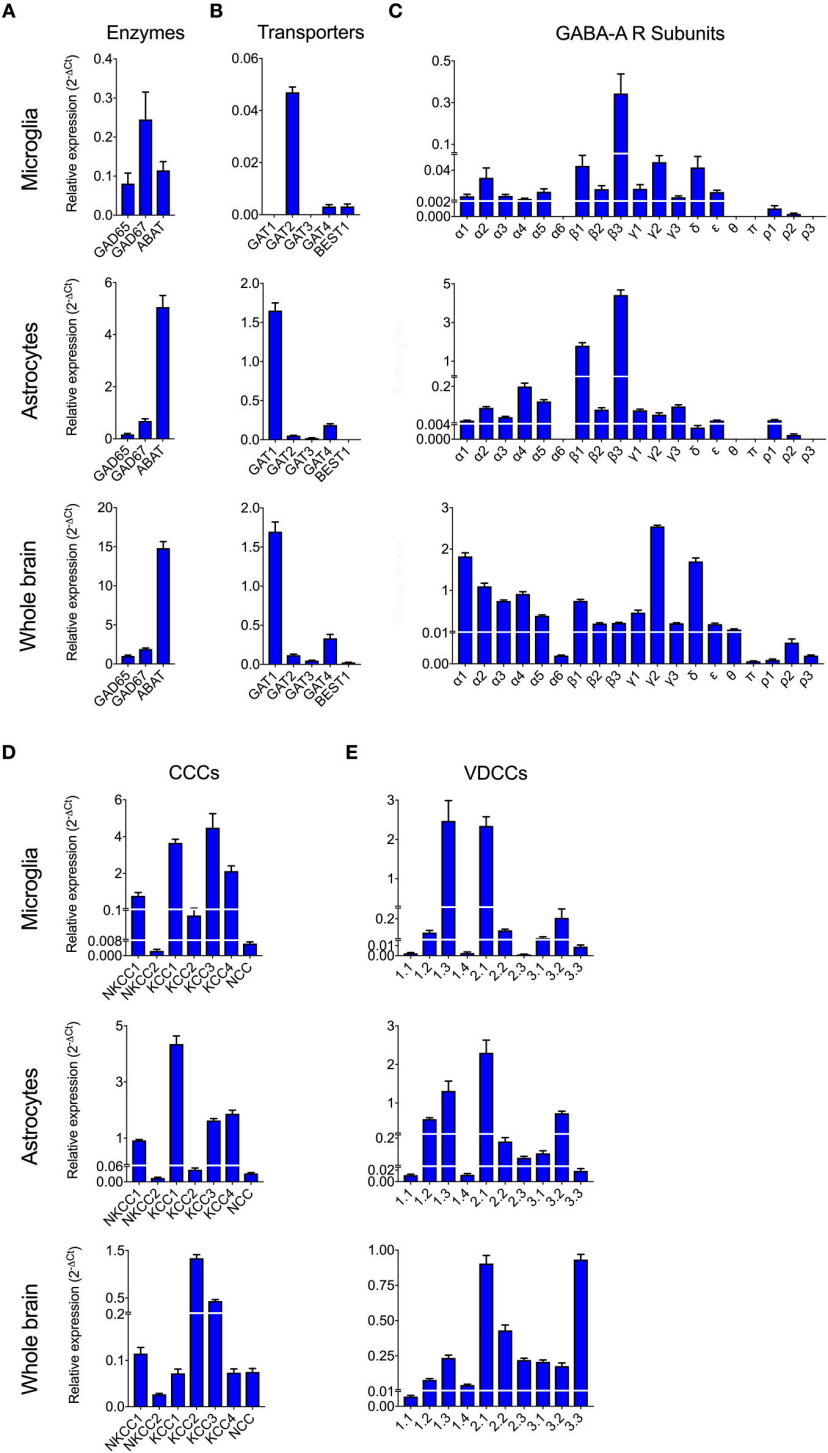
Expression of GABAergic and VDCC signaling components by microglia. The mRNA expression was analyzed by standard qPCR assay as indicated under Materials and Methods. The ΔCt values for each target were calculated relative to a normalization factor. The relative expression (2^−Δ*Ct*^) is represented as bar graphs with mean + SEM of 6 independent experiments for microglia, 5 for astrocytes and 4 for brain samples. The mRNA expression levels for **(A)** GABA synthesis and degrading enzymes, **(B)** GABA transporters, **(C)** GABA-A Receptor (R) subunits, **(D)** Cation Chloride co-transporters (CCCs), and **(E)** VDCCs are shown in unchallenged primary microglia, astrocytes, and whole brain.

**Figure 3:**
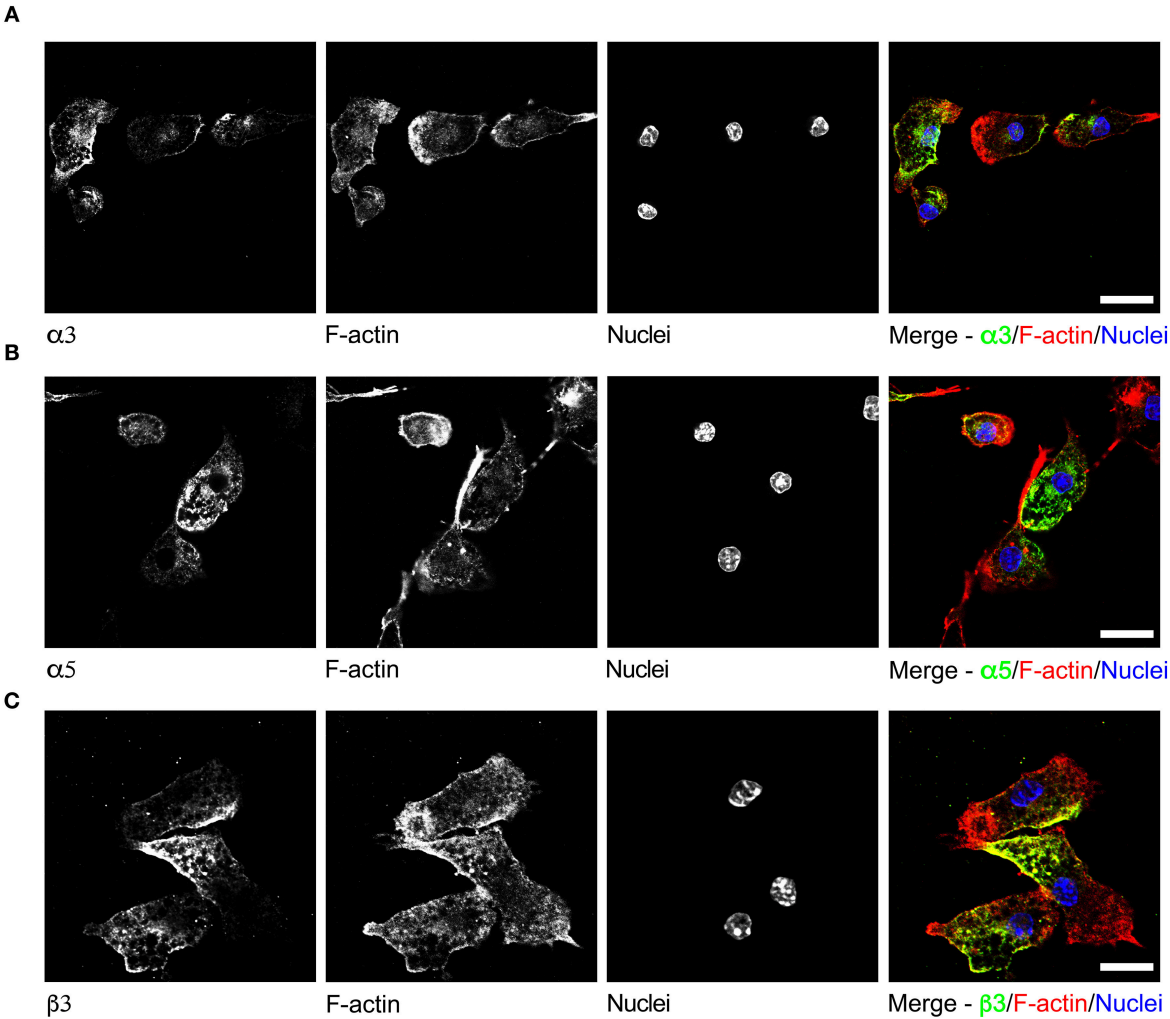
Immunostaining of GABA-A R subunits in primary microglia. Representative micrographs of unchallenged primary microglia stained with antibodies against GABA-A R **(A)** α3 subunit, **(B)** α5 subunit, and **(C)** β3 subunit, respectively, together with Alexa Fluor 647 Phalloidin (F-actin) and DAPI (nuclei), as indicated under Materials and Methods. Representative data is shown from 4 independent experiments. Scale bars, for **(A,B)** = 10 μm, for **(C)** = 5 μm.

To date, GABAergic signaling in microglia has remained chiefly unexplored (Barragan et al., 2015). The implication of microglia during toxoplasmic encephalitis (Dellacasa-Lindberg et al., 2011) and the participation of GABAergic/VDCC signaling in hypermigration of *Toxoplasma*-infected DCs (Fuks et al., 2012; Kanatani et al., 2017) motivated an assessment of GABAergic and VDCC signaling components in microglia. First, microglia and astrocyte preparations were characterized by transcriptional expression of a panel of markers. A prominent differential expression of Iba1, CD11b (microglia markers) and GFAP, GLT1, Aquaporin 4 (astrocyte markers) was detected (Figure S1 and Table S1). Second, a transcriptional analysis of primary cortical microglia revealed presence of mRNAs for (i) GABA enzymes GAD65, GAD67, and GABA-T (Figure 2A), (ii) GABA transporters GAT2, GAT4, and bestrophin 1 (BEST1) (Figure 2B), (iii) 15 GABA-A R subunits (α1-5, β1-3, γ1-3, δ, ε, ρ1-2) (Figure 2C), (iv) CCCs including NKCC1-2, KCC1-4, and NCC (Figure 2D), and (v) 10 VDCC Ca_V_ subunits (Figure 2E). Additionally, the expression of these components was assessed in astrocytes and whole brain (Figures 2A–E). All the components analyzed were detected in the whole brain samples. Further, an analysis of the relative expression in astrocytes and microglia revealed differential expression of GABA-T, GAT1, GAT3, GAT4, BEST1, GABA-A R subunits α4, β1, and δ, and a VDCC channel Ca_V_ 2.3 (Table S3). Two of the GABA transporters, GAT1 and GAT3, were undetectable in microglia whereas BEST1 was undetectable in astrocytes. Additionally, the relative quantitative expression of GABA-T, GAT4, Ca_V_ 2.3, α4, and β1 GABA-A R subunits was ~40- to 60-fold higher in astrocytes whereas the expression of the δ GABA-A R subunit was ~14-fold higher in microglia compared with astrocytes (Table S3). In general, microglia exhibited a broad expression of GABA-A R subunits with predominance of the β3 subunit (Figure 2C), similar to the expression profile of astrocytes. In addition, immunocytochemical stainings were consistent with protein expression of the α3, α5, and β3 GABA-A R subunits in microglia (Figure 3). A similar setup of CCCs and VDCCs was transcribed in microglia, astrocytes, and brain (Figures 2D,E), advocating for constitutive roles in these cells and brain tissue. Overall, we conclude that primary microglia transcriptionally express the necessary components to form functional GABAergic and VDCC signaling systems.

### Challenge With *T. gondii* Modulates the Expression GABAergic and VDCC Signaling Components in Microglia

**Figure 4:**
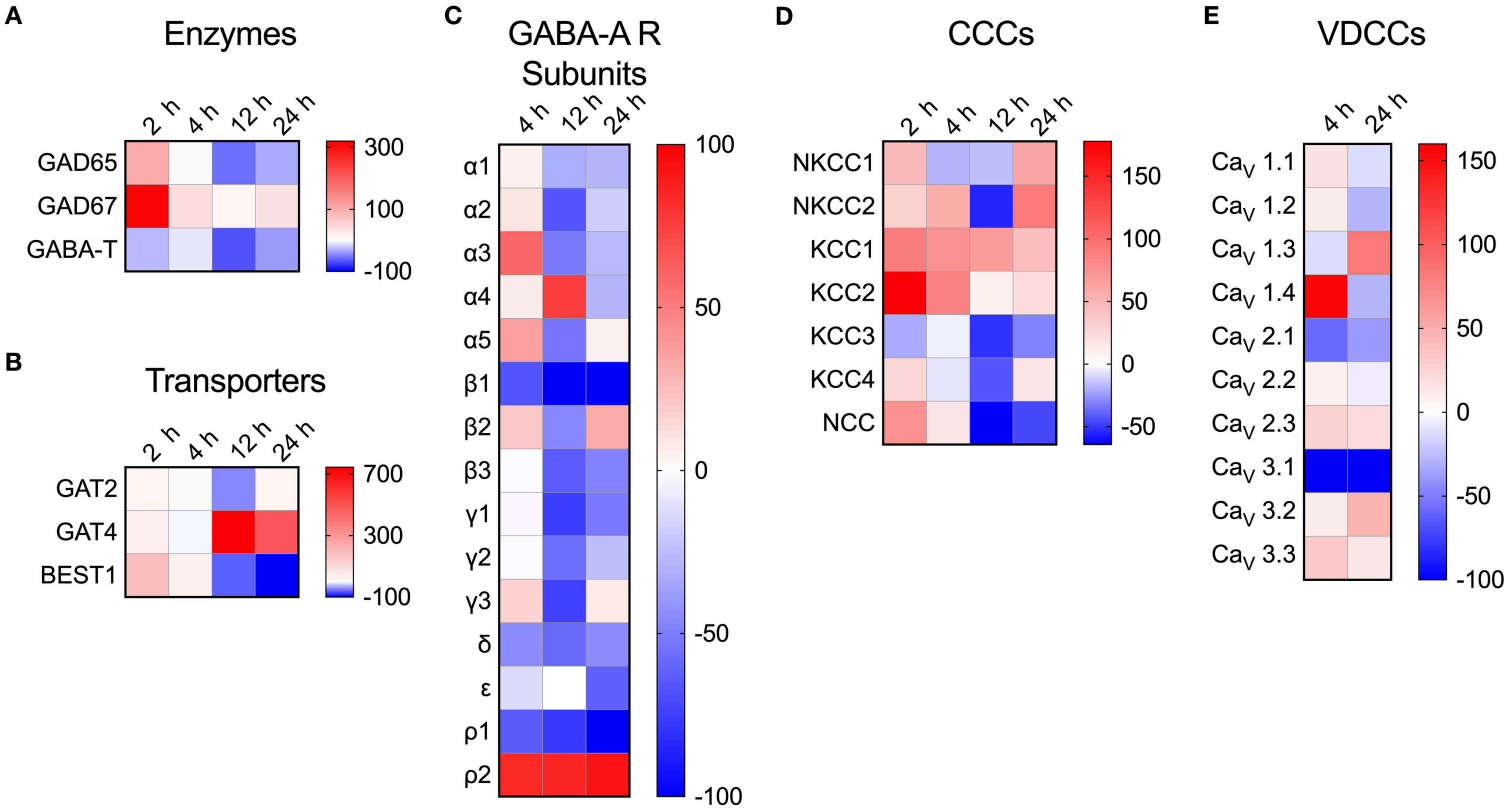
Modulated transcriptional expression of GABAergic and VDCC signaling components in *Toxoplasma*-challenged primary microglia. The relative expression (2^−Δ*Ct*^) for each target gene in *Toxoplasma* (PTG)-challenged primary microglia was normalized to that of in unchallenged microglia and the differences are represented as percentage increase (red color intensity scale) or decrease (blue scale) in the heat map. For both conditions, unchallenged and *Toxoplasma*-challenged, data is represented as mean of 2–4 independent experiments at indicated time points. The modulation of mRNA expression is shown for **(A)** GABA synthesis and degrading enzymes, **(B)** GABA transporters, **(C)** GABA-A R subunits, **(D)** CCCs, and **(E)** VDCCs.

To address the impact of *T. gondii* infection on the microglial GABAergic and VDCC signaling components, the mRNA expression was assessed at 2, 4, 12, and 24 h post-infection and related to unchallenged microglia at the corresponding time point. The data revealed modulated expression of multiple components. Shortly after *Toxoplasma* challenge, microglia upregulated the expression of the GABA synthesis enzymes GAD65/67 while expression of the GABA degradation enzyme GABA-T was downregulated (Figure 4A and Table S4). Additionally, expression of the GABA transporter GAT4 was upregulated (Figure 4B and Table S4). Jointly, these changes are all in theory consistent with elevated GABA concentrations. Further, additional components modulated (>50% up- or down) by *Toxoplasma* challenge included: (i) GABA-A R subunits α2-5, β1, β3, γ1-3, δ, ρ1, and ρ2 (Figure 4C and Table S4), (ii) CCCs NKCC1-2, KCC1-3, and NCC (Figure 4D and Table S4), and (iii) VDCCs Ca_V_1.3, 1.4, 2.1, and 3.1 (Figure 4E and Table S4). Thus, *Toxoplasma* infection modulates the expression of components of the GABAergic and VDCC signaling systems in microglia.

### Implication of the GABAergic and VDCC Signaling Systems in *Toxoplasma*-Induced Hypermotility of Microglia

**Figure 5:**
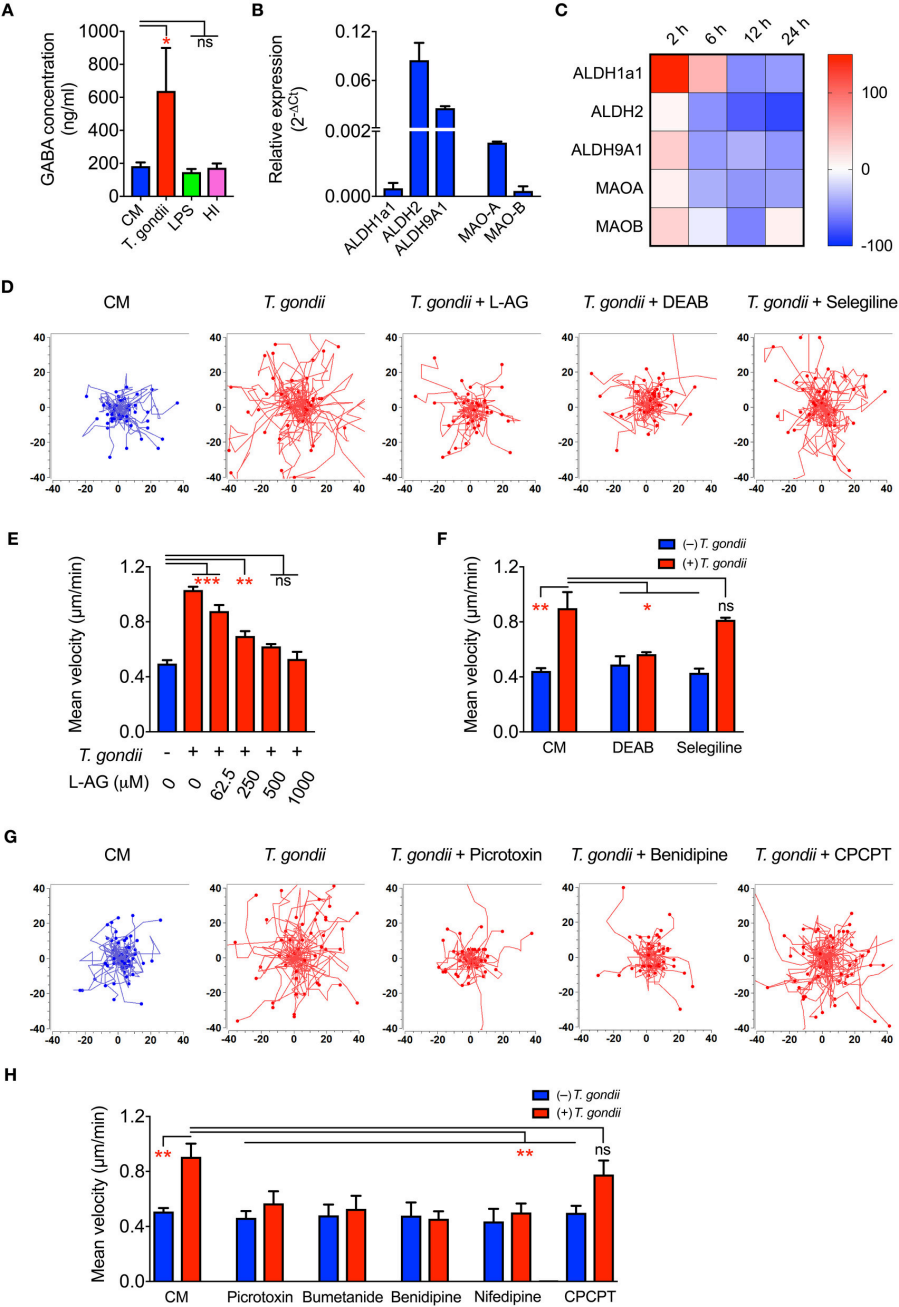
GABA secretion and impact of GABAergic inhibitors in *Toxoplasma-*challenged microglia. **(A)** GABA concentration in the supernatants from microglia incubated with complete medium (CM), PRU tachyzoites, LPS (100 ng/ml) or heat-inactivated tachyzoites (HI) was quantified by ELISA as indicated under Materials and Methods. **(B)** The relative mRNA expression (2^−Δ*Ct*^) of the enzymes -Aldehyde dehydrogenases (ALDH1a1, ALDH2, ALDH9A1) and monoamine oxidases (MAO-A, MAO-B) was analyzed in unchallenged microglia as indicated under Materials and Methods. **(C)** Modulation of enzymes indicated in **(B)** in *Toxoplasma*-challenged microglia in comparison to unchallenged microglia is presented as percentage increase (red color intensity scale) or decrease (blue scale) in a heat map at indicated time points. **(D)** Representative motility plots of microglia incubated with PRU tachyzoites and treated for 4 h with L-allylglycine (L-AG), DEAB, and selegiline as indicated under Materials and Methods. **(E)** Mean velocities of unchallenged and *Toxoplasma*-challenged microglia, as in **(D)**, treated with GAD inhibitor L-allylglycine (L-AG) at concentrations ranging from 62.5 to 1,000 μM. **(F)** Mean velocities of unchallenged and *Toxoplasma*-challenged microglia, as in **(D)**, incubated with complete medium (CM), DEAB (10 μM) or selegeline (10 μM). **(G)** Representative motility plots of microglia incubated with PRU tachyzoites and treated for 4 h with picrotoxin, benidipine or CPCPT. **(H)** Mean velocities of unchallenged and *Toxoplasma*-challenged microglia incubated with complete medium (CM), picrotoxin (50 μM), bumetanide (10 μM), benidipine (10 μM), nifedipine (10 μM), or CPCPT (1 μM). For **(A,B,E,F,H)**, bar graphs represent mean + SEM from 3 independent experiments. For **(A)**, statistical significance was tested by Student's *t*-test and for **(E,F,H)**, by One-Way ANOVA with Dunnett's *post-hoc* test. ns *p* ≥ 0.05, ^*^p < 0.05, ^**^p < 0. 01, ^***^p < 0. 001.

Upon *Toxoplasma* challenge of microglia, we observed transcriptional upregulation of GABA synthesis enzymes GAD65/67 and GABA transporters, with down-modulation of GABA-degrading enzyme GABA-T. To determine if this, in fact, translated into elevation of GABA, we assessed secretion of GABA in cell supernatants. Importantly, both primary microglia and BV2 microglia cells challenged with *T. gondii* tachyzoites exhibited significantly elevated GABA concentrations in the supernatant, contrasting non-significant effects upon challenge with heat-inactivated tachyzoites or LPS (Figure 5A and Figure S2E). In addition to the enzymes GAD65/67 that constitute the conventional GABA synthesis pathway, the mRNA for other enzymes, MAO-B, ALDH2, and ALDH1a1, known to synthesize GABA from putrescine by an alternative pathway in astrocytes and neurons, were also detected in primary microglia (Figure 5B). Additionally, *T. gondii* infection modulated their expression (Figure 5C).

Further, direct pharmacological inhibition of the GABA synthesis enzymes, GAD65/67 by L-allylglycine (Figures 5D,E) and ALDH1a1 and ALDH2 by DEAB (Figures 5D,F) abolished hypermotility, whereas an inhibitor of MAO-B (selegeline, acting upstream of ALDH2/ALDH1a1) non-significantly reduced hypermotility (Figures 5D,F). This indicated a putative activation of both the conventional and the alternative pathway for GABA synthesis (Kim et al., 2015) and the implication of GABA in the induction of hypermotility in *Toxoplasma*-infected microglia.

Next, we assessed the implication of GABA-A Rs and VDCCs by applying pharmacological antagonism. Indeed, antagonism of (i) GABA-A R by the direct channel pore blocker picrotoxin, (ii) NKCC1, a regulator of GABA-A R, by bumetanide, and (iii) VDCCs by the broad inhibitor benidipine and L-type inhibitor, nifedipine abolished hypermotility, with non-significant effects on the base-line motility of unchallenged microglia (Figures 5G,H). In contrast to DCs (Kanatani et al., 2017), the Ca_V_1.3 inhibitor CPCPT non-significantly impacted the hypermotility of *Toxoplasma*-infected microglia (Figures 5G,H). We conclude that, upon *Toxoplasma* challenge, microglia secrete GABA and that inhibition of GABAergic signaling by targeting GABA synthesis, GABA-A R antagonism or regulator antagonism, and VDCC antagonism abolishes hypermotility.

## Discussion

Previous work has established that *T. gondii* tachyzoites induce a hypermigratory phenotype in parasitized DCs and monocytic cells (Lambert et al., 2006; Kanatani et al., 2017; Cook et al., 2018). Yet, the impact of *Toxoplasma* infection on the migratory functions of glial cells, that mediate immune surveillance in the CNS, has remained unknown.

Our data demonstrate that a migratory activation of primary cortical microglia sets in upon *T. gondii* infection, in both 2D and 3D matrix confinements. The onset of high-velocity locomotion was accompanied by the dissolution of adhesion-mediating podosome structures and the acquisition of rounded cell morphology. Thus, parasitized microglia acquired morphological characteristics and locomotion that are reminiscent of the high-speed amoeboid migration mode of activated DCs (Lämmermann et al., 2008) and of hypermotile *Toxoplasma*-infected DCs (Weidner et al., 2013; Kanatani et al., 2015). Interestingly, similar morphological changes and migratory activation were confirmed in the microglia cell line BV2 but were absent in primary astrocytes. In spite that primary microglia and astrocytes are similarly permissive to *Toxoplasma* infection *in vitro* (Dellacasa-Lindberg et al., 2011), this underlines that the hypermigratory response differs between the two cell types. The data are in line with the previously observed enhanced transmigration by *Toxoplasma*-infected microglia, but not by astrocytes (Dellacasa-Lindberg et al., 2011) and also differences among leukocyte types (Lambert et al., 2011). Also, microglia are highly migratory cells in response to inflammatory cues while astrocytes have additional important structural functions in the CNS (Sofroniew and Vinters, 2010). Further, the finding that LPS, supernatants from infected microglia or heat-inactivated tachyzoites were unable to induce hypermotility in microglia indicates that the migratory activation was linked to the presence of intracellular *T. gondii*. In inflammatory conditions, highly migratory amoeboid microglial cells (AMC) have been reported (Deng et al., 2009). However, contrasting with the classical activation observed for AMC, a down-modulation of activation markers, e.g., MHC II and CD86, was observed in *Toxoplasma*-infected microglia (Dellacasa-Lindberg et al., 2011). This indicated that alternative activation mechanism(s) lied behind *Toxoplasma*-induced hypermotility.

We report that GABAergic signaling is implicated in the *Toxoplasma*-induced hypermotility of primary cortical microglia. In relation to neurotransmission, the GABAergic systems of neurons and astrocytes have been extensively studied (Lee et al., 2011; Kilb, 2012). However, because chiefly immune surveillance functions have been attributed to microglia, their GABAergic potential has remained unexplored (Barragan et al., 2015). Human microglia have been previously reported to express GABA-Transaminase and 3 GABA-A R subunits (α1, α3, and β1), indicating GABAceptive functions (Lee et al., 2011). Notably, we report that murine primary microglia secrete GABA upon *Toxoplasma* challenge, indicating they are GABAergic cells. Additionally, microglia transcriptionally expressed GABA synthesis and degradation enzymes, GABA transporters and GABA-A R subunits, all consistent with the existence of a GABAergic system.

We show that both primary cortical microglia and a microglia cell line (BV2) respond to *Toxoplasma* infection with GABA secretion and hypermotility. Primary microglia transcriptionally expressed the enzymes that constitute the conventional pathway of GABA synthesis (GAD 65/67) and upon challenge with *Toxoplasma*, both GAD65 and GAD67 mRNA exhibited a significant up-regulation (~2- to 4-fold). Somewhat in contrast, only GAD65 mRNA was detected in infected DCs (Fuks et al., 2012) and the mRNA of the GABA catabolic enzyme ABAT was more strongly expressed in astrocytes compared with microglia (~44-fold difference). Jointly, this is indicative of differences in GABA metabolism between the two cell types. Further, pharmacological inhibition of GAD65/67 inhibited *Toxoplasma*-induced hypermotility in microglia but required high concentrations of the inhibitor. However, pharmacological inhibition of the alternative pathway enzymes ALDH2/ALDH1a1 also inhibited hypermotility. Jointly, this advocates that the conventional and alternative GABA synthesis pathways cooperate in GABA production in microglia and that GABA synthesis is necessary to maintain hypermotility, as previously demonstrated in DCs (Fuks et al., 2012; Kanatani et al., 2017). Also, similar to observations in DCs, supernatants from infected microglia (containing secreted GABA) were insufficient to induce hypermigration of naïve microglia. This is also in line with the concept that a live intracellular tachyzoite is necessary to trigger the hypermigratory phenotype (Fuks et al., 2012; Kanatani et al., 2017) and may indicate that prior GABA-A R activation is necessary.

Another major difference between microglia, astrocytes, and DCs was the transcriptional expression of GABA transporters. While microglia preferentially expressed GAT2 (and GAT1 was undetectable), astrocytes preferentially expressed GAT1 (while BEST1 was undetectable). Additionally, microglia expressed the GABA regulators CCCs and VDCCs, with modulated expression upon *Toxoplasma* challenge.

Further, the subunit repertoire of transcriptionally expressed GABA-A R subunits by microglia was broader than that of myeloid DCs (Fuks et al., 2012) and remarkably similar to the repertoire expressed by astrocytes, despite the different ontogenic origins of these two cell types (Hochstim et al., 2008). However, quantitative differences were present. For example, the δ subunit exhibited ~14-fold higher relative expression in microglia while the α4 and β1 subunits exhibited ~40- to 60-fold higher relative expression in astrocytes. A caveat of the purification protocol is always the possibility of contaminating astrocyte-derived mRNA in microglia purifications and vice versa. However, quantitative analyses based on transcription of classical microglia and astrocyte markers showed that this contamination should be a minor contributor and cannot explain the overall qualitative and quantitative expression differences between microglia and astrocytes. Jointly, the data show that microglia are GABAergic cells. Future research needs to determine how GABA receptors and other GABAergic components traffic in the infected cell and become activated and/or modulated by the infection.

Importantly, pharmacological antagonism targeting various levels of the GABAergic motogenic axis, i.e., GABA synthesis, GABA-A R antagonism, GABA-A R regulator or VDCC antagonism, abolished *Toxoplasma*-induced hypermotility, concordant with results in DCs (Fuks et al., 2012; Kanatani et al., 2017). However, differences also exist. For example, a specific inhibitor of the VDCC subtype Ca_V_1.3 abolished the *Toxoplasma*-induced hypermotility of DCs (Kanatani et al., 2017) but non-significantly impacted on the hypermotility of microglia. This corresponds well with the predominant expression of Ca_V_1.3 over other VDCC subtypes in DCs and the broader expression, of Ca_V_1.3 but also additional VDCC subtypes, in cortical microglia. Jointly, the data underline the functional implication of GABAergic signaling in *Toxoplasma*-induced hypermigration of primary microglia.

Additionally, the parasite-derived 14-3-3 molecule has been linked to the hypermigratory phenotype in both DCs and microglia (BV2), with sequestration of host-cell 14-3-3 (Weidner et al., 2016). 14-3-3 is an abundant molecule in the CNS (Sluchanko and Gusev, 2010) that can regulate GABA receptor function (Laffray et al., 2012). Thus, future research needs to address how 14-3-3 is implicated in *Toxoplasma*-induced GABA-mediated hypermotility of microglia.

The finding that *Toxoplasma* infection induces GABA secretion by microglia raises additional questions in relation to infection in the CNS. As the neurotransmitter systems are tightly regulated, any changes in the expression of components or the regulation of these systems might lead to altered balance between excitation and inhibition in the CNS (Semyanov et al., 2004). Our data are well in line with recent descriptions of GABAergic dysregulation (Brooks et al., 2015) and glutamatergic dysregulation (David et al., 2016) in murine neurotoxoplasmosis. Hypothetically, local elevations of GABA concentrations by *Toxoplasma* infection may alter the neuronal functions, with impacts on normal brain physiology and altered host behavior (Brooks et al., 2015). Additionally, GABA has been attributed chemokinetic and chemotactic effects on migrating embryonic neurons (Behar et al., 1996). In DCs, a GABA-mediated motogenic effect is observed but no chemotactic effect was detected (Fuks et al., 2012). Of note is that astrocytes, despite expressing GABA receptors and secreting GABA, did not respond with hypermotility upon *Toxoplasma* infection. This may be due to differential composition of subunits or differential activation profile (and therefore functionality) of GABA receptors or due to differences in signaling downstream of GABA receptor activation, e.g., signal transduction via VDCCs and other downstream signaling pathways (Kanatani et al., 2017). Finally, mounting evidence implicates GABA in the immune functions of T cells and DCs (Barragan et al., 2015). This raises the question whether GABA should be considered a “*neuro-immuno-transmitter*,” as recently suggested for dopamine (Levite, 2016).

Based on the data at hand, we hypothesize that the induced hypermigration of infected microglia may facilitate parasite dispersion in the brain parenchyma. The findings also open up for the speculation that GABA secretion by *Toxoplasma*-infected glial cells may have migratory effects of surrounding cells in the parenchymal microenvironment and modulate GABA levels locally leading to altered neuronal functions. Finally, leukocytes have been implicated in the delivery of *Toxoplasma* to the brain parenchyma (Courret et al., 2006) with rapid parasite transfer between microglia and T cells (Dellacasa-Lindberg et al., 2011). Thus, the production of GABA by infected cells could hypothetically serve as a signal attracting new host cells and thereby facilitating dissemination within the parenchyma. These alternatives need to be explored and constitute novel perspectives on the pathogenesis of toxoplasmic encephalitis.

## Data Availability

All datasets generated for this study are included in the manuscript and/or the supplementary files.

## Ethics Statement

The Regional Animal Research Ethical Board, Stockholm, Sweden, approved protocols involving extraction of cells from mice, following proceedings described in EU legislation (Council Directive 2010/63/EU).

## Author Contributions

AKB and SK performed experiments and analyzed data. AKB, SK, and AB conceived experimental design and wrote the manuscript.

### Conflict of Interest Statement

The authors declare that the research was conducted in the absence of any commercial or financial relationships that could be construed as a potential conflict of interest.

## References

[B1] Azuma H. Inamoto T. Sakamoto T. Kiyama S. Ubai T. Shinohara Y.. (2003). Gamma-aminobutyric acid as a promoting factor of cancer metastasis; induction of matrix metalloproteinase production is potentially its underlying mechanism. Cancer Res. 63, 8090–8096. 14678958

[B2] Barragan A. Weidner J. M. Jin Z. Korpi E. R. Birnir B. (2015). GABAergic signalling in the immune system. Acta Physiol. 213, 819–827. 10.1111/apha.12467 25677654

[B3] Behar T. N. Li Y. X. Tran H. T. Ma W. Dunlap V. Scott C.. (1996). GABA stimulates chemotaxis and chemokinesis of embryonic cortical neurons via calcium-dependent mechanisms. J. Neurosci. 16, 1808–1818. 8774448 10.1523/JNEUROSCI.16-05-01808.1996 PMC6578698

[B4] Bhandage A. K. Barragan A. (2019). Calling in the CaValry - *Toxoplasma gondii* hijacks GABAergic signaling and voltage-dependent calcium channel signaling for Trojan horse-mediated dissemination. Front. Cell. Infect. Microbiol. 2019:61 10.3389/fcimb.2019.00061 PMC6436472 30949456

[B5] Brooks J. M. Carrillo G. L. Su J. Lindsay D. S. Fox M. A. Blader I. J. (2015). *Toxoplasma gondii* infections alter GABAergic synapses and signaling in the central nervous system. MBio 6:e01428-15. 10.1128/mBio.01428-15. 26507232 PMC4626855

[B6] Carruthers V. B. Suzuki Y. (2007). Effects of *Toxoplasma gondii* infection on the brain. Schizophr. Bull. 33, 745–751. 10.1093/schbul/sbm008 17322557 PMC2526127

[B7] Cook J. H. Ueno N. Lodoen M. B. (2018). *Toxoplasma gondii* disrupts beta1 integrin signaling and focal adhesion formation during monocyte hypermotility. J. Biol. Chem. 293, 3374–3385. 10.1074/jbc.M117.793281 29295815 PMC5836128

[B8] Courret N. Darche S. Sonigo P. Milon G. Buzoni-Gatel D. Tardieux I. (2006). CD11c- and CD11b-expressing mouse leukocytes transport single *Toxoplasma gondii* tachyzoites to the brain. Blood 107, 309–316. 10.1182/blood-2005-02-0666 16051744 PMC1895351

[B9] David C. N. Frias E. S. Szu J. I. Vieira P. A. Hubbard J. A. Lovelace J.. (2016). GLT-1-dependent disruption of CNS glutamate homeostasis and neuronal function by the protozoan parasite *Toxoplasma gondii*. PLoS Pathog. 12:e1005643. 10.1371/journal.ppat.1005643 27281462 PMC4900626

[B10] Dellacasa-Lindberg I. Fuks J. M. Arrighi R. B. Lambert H. Wallin R. P. Chambers B. J.. (2011). Migratory activation of primary cortical microglia upon infection with *Toxoplasma gondii*. Infect. Immun. 79, 3046–3052. 10.1128/IAI.01042-10 21628522 PMC3147544

[B11] Deng Y. Y. Lu J. Ling E. A. Kaur C. (2009). Monocyte chemoattractant protein-1 (MCP-1) produced via NF-kappaB signaling pathway mediates migration of amoeboid microglia in the periventricular white matter in hypoxic neonatal rats. Glia 57, 604–621. 10.1002/glia.20790 18942743

[B12] Frénal K. Dubremetz J. F. Lebrun M. Soldati-Favre D. (2017). Gliding motility powers invasion and egress in Apicomplexa. Nat. Rev. Microbiol. 15, 645–660. 10.1038/nrmicro.2017.86 28867819

[B13] Fuks J. M. Arrighi R. B. Weidner J. M. Kumar Mendu S. Jin Z. Wallin R. P.. (2012). GABAergic signaling is linked to a hypermigratory phenotype in dendritic cells infected by *Toxoplasma gondii*. PLoS Pathog. 8:e1003051. 10.1371/journal.ppat.1003051 23236276 PMC3516538

[B14] Ginhoux F. Greter M. Leboeuf M. Nandi S. See P. Gokhan S.. (2010). Fate mapping analysis reveals that adult microglia derive from primitive macrophages. Science 330, 841–845. 10.1126/science.1194637 20966214 PMC3719181

[B15] Hochstim C. Deneen B. Lukaszewicz A. Zhou Q. Anderson D. J. (2008). Identification of positionally distinct astrocyte subtypes whose identities are specified by a homeodomain code. Cell 133, 510–522. 10.1016/j.cell.2008.02.046 18455991 PMC2394859

[B16] Höglund P. J. Adzic D. Scicluna S. J. Lindblom J. Fredriksson R. (2005). The repertoire of solute carriers of family 6: identification of new human and rodent genes. Biochem. Biophys. Res. Commun. 336, 175–189. 10.1016/j.bbrc.2005.08.048 16125675

[B17] Joynson D. H. Wreghitt T. J. (2001). Toxoplasmosis: A Comprehensive Clinical Guide. Cambridge: Cambridge University Press.

[B18] Kahle K. T. Staley K. J. Nahed B. V. Gamba G. Hebert S. C. Lifton R. P.. (2008). Roles of the cation-chloride cotransporters in neurological disease. Nat. Clin. Pract. Neurol. 4, 490–503. 10.1038/ncpneuro0883 18769373

[B19] Kanatani S. Fuks J. M. Olafsson E. B. Westermark L. Chambers B. Varas-Godoy M.. (2017). Voltage-dependent calcium channel signaling mediates GABAA receptor-induced migratory activation of dendritic cells infected by *Toxoplasma gondii*. PLoS Pathog. 13:e1006739. 10.1371/journal.ppat.1006739 29216332 PMC5720541

[B20] Kanatani S. Uhlen P. Barragan A. (2015). Infection by *Toxoplasma gondii* induces amoeboid-like migration of dendritic cells in a three-dimensional collagen matrix. PLoS ONE 10:e0139104. 10.1371/journal.pone.0139104 26406763 PMC4583262

[B21] Kilb W. (2012). Development of the GABAergic system from birth to adolescence. Neuroscientist 18, 613–630. 10.1177/1073858411422114 21952258

[B22] Kim J. I. Ganesan S. Luo S. X. Wu Y. W. Park E. Huang E. J.. (2015). Aldehyde dehydrogenase 1a1 mediates a GABA synthesis pathway in midbrain dopaminergic neurons. Science 350, 102–106. 10.1126/science.aac4690 26430123 PMC4725325

[B23] Kreutzberg G. W. (1996). Microglia: a sensor for pathological events in the CNS. Trends Neurosci. 19, 312–318. 8843599 10.1016/0166-2236(96)10049-7

[B24] Laffray S. Bouali-Benazzouz R. Papon M. A. Favereaux A. Jiang Y. Holm T.. (2012). Impairment of GABAB receptor dimer by endogenous 14-3-3zeta in chronic pain conditions. EMBO J. 31, 3239–3251. 10.1038/emboj.2012.161 22692127 PMC3411072

[B25] Lambert H. Dellacasa-Lindberg I. Barragan A. (2011). Migratory responses of leukocytes infected with *Toxoplasma gondii*. Microbes Infect. 13, 96–102. 10.1016/j.micinf.2010.10.002 20951223

[B26] Lambert H. Hitziger N. Dellacasa I. Svensson M. Barragan A. (2006). Induction of dendritic cell migration upon *Toxoplasma gondii* infection potentiates parasite dissemination. Cell. Microbiol. 8, 1611–1623. 10.1111/j.1462-5822.2006.00735.x 16984416

[B27] Lambert H. Vutova P. P. Adams W. C. Lore K. Barragan A. (2009). The *Toxoplasma gondii*-shuttling function of dendritic cells is linked to the parasite genotype. Infect. Immun. 77, 1679–1688. 10.1128/IAI.01289-08 19204091 PMC2663171

[B28] Lämmermann T. Bader B. L. Monkley S. J. Worbs T. Wedlich-Soldner R. Hirsch K.. (2008). Rapid leukocyte migration by integrin-independent flowing and squeezing. Nature 453, 51–55. 10.1038/nature06887 18451854

[B29] Lee M. Schwab C. McGeer P. L. (2011). Astrocytes are GABAergic cells that modulate microglial activity. Glia 59, 152–165. 10.1002/glia.21087 21046567

[B30] Levite M. (2016). Dopamine and T cells: dopamine receptors and potent effects on T cells, dopamine production in T cells, and abnormalities in the dopaminergic system in T cells in autoimmune, neurological and psychiatric diseases. Acta Physiol. 216, 42–89. 10.1111/apha.12476 25728499

[B31] Lüder C. G. Giraldo-Velasquez M. Sendtner M. Gross U. (1999). *Toxoplasma gondii* in primary rat CNS cells: differential contribution of neurons, astrocytes, and microglial cells for the intracerebral development and stage differentiation. Exp. Parasitol. 93, 23–32. 10464035 10.1006/expr.1999.4421

[B32] Nebuloni M. Pellegrinelli A. Ferri A. Tosoni A. Bonetto S. Zerbi P.. (2000). Etiology of microglial nodules in brains of patients with acquired immunodeficiency syndrome. J. Neurovirol. 6, 46–50. 10786996 10.3109/13550280009006381

[B33] Nimmerjahn A. Kirchhoff F. Helmchen F. (2005). Resting microglial cells are highly dynamic surveillants of brain parenchyma *in vivo*. Science 308, 1314–1318. 10.1126/science.1110647 15831717

[B34] Olsen R. W. Sieghart W. (2008). International Union of Pharmacology. LXX. Subtypes of gamma-aminobutyric acid(A) receptors: classification on the basis of subunit composition, pharmacology, and function. Update Pharmacol. Rev. 60, 243–260. 10.1124/pr.108.00505 18790874 PMC2847512

[B35] Scheidegger A. Vonlaufen N. Naguleswaran A. Gianinazzi C. Muller N. Leib S. L.. (2005). Differential effects of interferon-gamma and tumor necrosis factor-alpha on *Toxoplasma gondii* proliferation in organotypic rat brain slice cultures. J. Parasitol. 91, 307–315. 10.1645/GE-379R 15986605

[B36] Seiler N. al-Therib M. J. Kataoka K. (1973). Formation of GABA from putrescine in the brain of fish (Salmo irideus Gibb.). J. Neurochem. 20, 699–708. 4703785 10.1111/j.1471-4159.1973.tb00030.x

[B37] Semyanov A. Walker M. C. Kullmann D. M. Silver R. A. (2004). Tonically active GABA A receptors: modulating gain and maintaining the tone. Trends Neurosci. 27, 262–269. 10.1016/j.tins.2004.03.005 15111008

[B38] Sluchanko N. N. Gusev N. B. (2010). 14-3-3 proteins and regulation of cytoskeleton. Biochem. Mosc. 75, 1528–1546. 10.1134/S0006297910130031 21417993

[B39] Sofroniew M. V. Vinters H. V. (2010). Astrocytes: biology and pathology. Acta Neuropathol. 119, 7–35. 10.1007/s00401-009-0619-8 20012068 PMC2799634

[B40] Soghomonian J. J. Martin D. L. (1998). Two isoforms of glutamate decarboxylase: why? Trends Pharmacol. Sci. 19, 500–505. 9871412 10.1016/s0165-6147(98)01270-x

[B41] Strack A. Asensio V. C. Campbell I. L. Schluter D. Deckert M. (2002). Chemokines are differentially expressed by astrocytes, microglia and inflammatory leukocytes in Toxoplasma encephalitis and critically regulated by interferon-gamma. Acta Neuropathol. 103, 458–468. 10.1007/s00401-001-0491-7 11935261

[B42] Suzuki Y. Claflin J. Wang X. Lengi A. Kikuchi T. (2005). Microglia and macrophages as innate producers of interferon-gamma in the brain following infection with *Toxoplasma gondii*. Int. J. Parasitol. 35, 83–90. 10.1016/j.ijpara.2004.10.020 15619519

[B43] Weidner J. M. Kanatani S. Hernandez-Castaneda M. A. Fuks J. M. Rethi B. Wallin R. P.. (2013). Rapid cytoskeleton remodelling in dendritic cells following invasion by *Toxoplasma gondii* coincides with the onset of a hypermigratory phenotype. Cell. Microbiol. 15, 1735–1752. 10.1111/cmi.12145. 23534541

[B44] Weidner J. M. Kanatani S. Uchtenhagen H. Varas-Godoy M. Schulte T. Engelberg K. (2016). Migratory activation of parasitized dendritic cells by the protozoan *Toxoplasma gondii* 14-3-3 protein. Cell. Microbiol. 2016:12595 10.1111/cmi.12595 PMC5040621 27018989

[B45] Wheeler D. W. Thompson A. J. Corletto F. Reckless J. Loke J. C. Lapaque N.. (2011). Anaesthetic impairment of immune function is mediated via GABA(A) receptors. PLoS ONE 6:e17152. 10.1371/journal.pone.0017152 21390329 PMC3044756

[B46] Wilson E. H. Hunter C. A. (2004). The role of astrocytes in the immunopathogenesis of toxoplasmic encephalitis. Int. J. Parasitol. 34, 543–548. 10.1016/j.ijpara.2003.12.010 15064118

[B47] Yoon B. E. Woo J. Chun Y. E. Chun H. Jo S. Bae J. Y.. (2014). Glial GABA, synthesized by monoamine oxidase B, mediates tonic inhibition. J. Physiol. 592, 4951–4968. 10.1113/jphysiol.2014.278754 25239459 PMC4259537

