# Supplementary material for: Toxoplasma-Induced Hypermigration of Primary Cortical Microglia Implicates GABAergic Signaling
Source: Front Cell Infect Microbiol. 2019 Mar 20;9:73. doi: 10.3389/fcimb.2019.00073 (PMC6436526; doi:10.3389/fcimb.2019.00073)
Supplement: Supplementary file 1 [file Table_1.pdf]

Table S1: Relative transcriptional expression of microglia and astrocyte markers in microglia and astrocyte cell preparations

	Relative expression (%)	
Markers [a]	Microglia	Astrocytes
Iba1	100 [b]	3.4
CD11b	100	6.0
HEXB	100	8.8
P2ry12	100	27.2
Cx3cr1	100	43.5
S100B	5.9	100 [c]
GFAP	0.5	100
GLT1	2.3	100
AQP4	0.6	100
ALDH1L1	9.5	100

[a] The relative mRNA expression of indicated markers was quantified in microglia and astrocyte cell preparations as described under Material and Methods.

[b] The mean expression of Iba1, CD11b, HEXB, P2ry12, Cx3cr1 was normalized to the expression levels in microglia cell samples (100%) and, respectively for each marker, the relative expression in astrocyte cell preparations was calculated. Data shows mean values (n = 5 samples per group).

[c] The mean expression of S100B, GFAP, GLT1, AQP4, ALDH1L1 was normalized to the expression levels in astrocyte cell samples (100%) and, respectively for each marker, the relative expression in microglia cell preparations was calculated. Data shows mean values (n = 5 samples per group)
