# Supplementary material for: Toxoplasma-Induced Hypermigration of Primary Cortical Microglia Implicates GABAergic Signaling
Source: Front Cell Infect Microbiol. 2019 Mar 20;9:73. doi: 10.3389/fcimb.2019.00073 (PMC6436526; doi:10.3389/fcimb.2019.00073)
Supplement: Supplementary file 2 [file Table_2.pdf]

Table S2: Primer pair sequences used for qPCR

mRNA target (*gene name*)	Forward primer sequence	Reverse primer sequence	Amplicon
Microglia/Astrocyte markers			
Iba1 (*Aif1*)	GGATTTGCAGGGAGGAAAAG	TGGGATCATCGAGGAATTG	92
P2Y12 (*P2ry12*)	CACGGATTCCCTACACCCTG	GGGTGCTCTCCTTCACGTAG	87
HEX-B (*Hexb*)	ACTCCAAGATTATGGCCTCGAGCA	AGCTATTCCACGGCTGACCATTCT	132
CD11b (*Itgam*)	GTGTGACTACAGCACAAGCCG	CCCAAGGACATATTCACAGCCT	75
CX3CR1 (*Cx3cr1*)	ACCGGTACCTTGCCATCGT	ACACCGTGCTGCACTGTCC	64
GFAP (*Gfap*)	CTCGTGTGGATTTGGAGAG	GTTCTCGAACTTCCTCCTC	87
GLT1 (*Slc1a2*)	AGTTTAATCACAGGGTTGTCAG	GGACATGTAATACACCATAGCTC	81
Aquaporin 4 (*AQP4*)	CTGATGTTACTGGTTCAATAGC	GGCTCCAGTATAATTGATTGC	83
S100Beta (*S100B*)	AAGCACAAGCTGAAGAAGTC	CTGCTCCTTGATTTCCTCC	81
ALDH1L1 (*Aldh1l1*)	TAGATGTTGTGAGGCTGGT	TGGCCATGTAAACATCCTC	81
Enxymes			
GAD65 (*Gad2*)	GCTGGAACCACCGTGTATGG	TCCACGTGCATCCAGATCTTAT	86
GAD67 (*Gad1*)	GTGACCAGGGTGCCCGCTTC	TGCGCAGTTTGCTCCTCCCC	100
GABA-T (*Abat*)	TGGCCTTCTTGTTGATTACC	TATATCTGGATCCAGGTATGAAGAG	81
Transporters			
GAT1 (*Slc6a1*)	TGAACTCTTCATTGCTGCC	AAGACATAAATGCCACCCTG	81
GAT2 (*Slc6a12*)	CATGATGCCTTTGTCCCAG	TACAGACGAACTGGCTGTC	83
GAT3 (*Slc6a13*)	ATCTTTGAAGGCATCGGCT	GAAGAGGTAGAAGAGGG	96
GAT4 (*Slc6a11*)	CATGCTGTGTATCCCACTC	GTCAACTTCTGTAATTTCTCGG	81
Bestrophin 1 (*Best1*)	CATCTACAAGCTGCTGTATGG	AGAACCATTCTGTAGAGTCCAC	81
GABA-A R subunits			
α1(*GabrA1*)	AAAAGCGTGGTTCCAGAAAA	GCTGGTTGCTGTAGGAGCAT	84
α2 (*GabrA2*)	GCTACGCTTACACAACCTCAGA	GACTGGCCCAGCAAATCATACT	115
α3 (*GabrA3*)	GCCGTCTGTTATGCCTTTGTATTT	TTCTTCATCTCCAGGGCCTCT	119

α4 (*GabrA4*)	AGAACTCAAAGGACGAGAAATTGT	TTCACTTCTGTAACAGGACCCC	118
α5 (*GabrA5*)	GATTGTGTTCCCCATCTTGTTTGGC	TTACTTTGGAGAGGTGGCCCCTTTT	100
α6 (*GabrA6*)	TGGGAGCTATGCTTATCCT	TCTTCTGGGACTTCTACTGAG	81
β1 (*GabrB1*)	GGTTTGTTGTGCACACAGCTCC	ATGCTGGCGACATCGATCCGC	153
β2 (*GabrB2*)	GCTGGTGAGGAAATCTCGGTCCC	CATGCGCACGGCGTACCAAA	70
β3 (*GabrB3*)	GAGCGTAAACGACCCCGGGAA	GGGACCCCCGAAGTCGGGTCT	100
γ1 (*GabrG1*)	ATCCACTCTCATTCCCATGAACAGC	ACAGAAAAAGCTAGTACAGTCTTTGC	100
γ2 (*GabrG2*)	ACTTCTGGTGACTATGTGGTGAT	GGCAGGAACAGCATCCTTATTG	147
γ3 (*GabrG3*)	ATTACATCCAGATTCCACAAGATG	CAC AGG TGT CCT CAA ATT CCT	149
δ (*GabrD*)	GAATCCGTTCCAGACTCAAA	GCACTAGGCTCAACTTCAGG	349
ε (*GabrE*)	ACTGCGCCCTGGCATTGGAG	AGGCCCGAGGCTGTTGACAA	70
θ (*GabrQ*)	GCTGGAGGTGGAGAGCTATGGCT	CCCCAGGTACGTGTACTGAGGGA	115
π (*GabrP*)	CAGAGGACGTGCATCCAGGGGA	TCCGAACTGGGTCACCACCGAA	139
ρ1 (*GabrR1*)	CTGGAAATCGAAAGCTACGC	AGATGTGACGACGCAGAGTG	205
ρ2 (*GabrR2*)	CCAAGCCAAGCCATTTGTAT	GTCCCTCCAGTAATGCCTCA	227
ρ3 (*GabrR3*)	CAACTCAACAGGAGGGGAAA	TCCACATCAGTCTCGCTGTC	101
CCCs			
NKCC1 (*Slc12a2*)	GATGCTGTGGTCGCATACACT	CAGCGGACTAATACACCCTTG	77
NKCC2 (*Slc12a1*)	CTGGCTAAGAATGTGACTGT	TCTGCTTGCTCATCTCCAG	83
KCC1 (*Slc12a4*)	TCTACCTGGGGACGACATTTG	CCGATGGGTAAAAGATGGCAG	102
KCC2 (*Slc12a5*)	TCAGTCACAGGGATCATGG	GGATAGTTCCAGTAGGGATAGAC	82
KCC3 (*Slc12a6*)	CTGCCATCTTTCGGAGTGACG	AGAAGGCTGTACCATAGACGC	81
KCC4 (*Slc12a7*)	ATGCCCACGAACTTTACGGTG	GGGAGGTTTGATCCACGCT	115
NCC (*Slc12a3*)	ACACGGCAGCACCTTATACAT	GAGGAATGAATGCAGGTCAGC	142
VDCCs			
CaV 1.1 (*Cacna1S*)	GGTAGCATGTAAGAGGCTG	GCAAATAGTGTGGCATTAAAGG	81
CaV 1.2 (*Cacna1C*)	CGTTCTCATCCTGCTCAACA	TATGCTCCCAATGACGATGA	208
CaV 1.3 (*Cacna1D*)	TGCACAGATGAAGCCAAAAG	GACCAACGTTCTCACCGTTT	229
CaV 1.4 (*Cacna1F*)	TCCATCATGAAGGCGCTTGT	CGAGTCCGATGATGGCGTAA	85

CaV 2.1 (*Cacna1A*)	AATTCCAAATCACGGAGCAC	CATCAGAAACGAGCACAGGA	218
CaV 2.2 (*Cacna1B*)	GCAACACATGGAACTGGTTG	GCATTCTTGTCCTCCTCTGC	238
CaV 2.3 (*Cacna1E*)	TGAAGGCTGTGTTTGACTGC	ATTCATGACGCTTCCATTCC	234
CaV 3.1 (*Cacna1G*)	TGTGGAAATGGTGGTGAAGA	ACTGCGGAGAAGCTGACATT	150
CaV 3.2 (*Cacna1H*)	TGGGAACGTGCTTCTTCTCT	GGGGATGTGTGAGCATTTCT	229
CaV 3.3 (*Cacna1I*)	GAACTCAGACAGGTCCTCG	CTAGCTCACCCTTGCTCTC	101
Other enzymes			
ALDH1A1 (*Aldh1a1*)	ACTGCTATATGATGTTGTCAGC	CCATGTTCACCCAGTTCTC	82
ALDH2 (*Aldh2*)	ATGTCTCCGCTATTACGCT	GTATAGCTGAAGAAGTCGCC	81
ALDH9A1 (*Aldh9a1*)	CTGGCGTGAAGATCATGGA	TGATGAGAGGAGATTTGCCC	81
MAO-A (*Maoa*)	ACATATACTGTGAGGAATGAGC	TAAGATTCTGTTCTGGGTTGG	81
MAO-B (*Maob*)	TATGGAATCCTATCACCTACCT	CATCACTGGGAATCTCTTGG	81
Reference genes			
TATA-binding protein (*TBP*)	GGGGAGCTGTGATGTGAAGT	CCAGGAAATAATTCTGGCTCA	93
Importin 8 (*IPO8*)	CTATGCTCTCGTTCAGTATGC	GTCCGAAAGATCTCCATCCA	81
Beta-Actin (*Actb*)	CACTGTCGAGTCGCGTCC	TCATCCATGGCGAACTGGTG	89
Glyceraldehyde-3-phosphate dehydrogenase (*GAPDH*)	TGACCTCAACTACATGGTCTACA	CTTCCCATTCTCGGCCTTG	85

Primer pair sequences are designed using GETprime or NCBI primer blast databases and used in real time qPCR as indicated in Materials and Methods.
