# Supplementary material for: Toxoplasma-Induced Hypermigration of Primary Cortical Microglia Implicates GABAergic Signaling
Source: Front Cell Infect Microbiol. 2019 Mar 20;9:73. doi: 10.3389/fcimb.2019.00073 (PMC6436526; doi:10.3389/fcimb.2019.00073)
Supplement: Supplementary file 3 [file Table_3.pdf]

Table S3: Relative transcriptional expression of GABAergic and VDCC components in astrocytes normalized to microglia

	**Normalized relative expression**[#]
**Enzymes**	
GAD65	2.1
GAD67	2.8
GABA-T	44.1

	**Normalized relative expression**[#]
**Transporters**	
GAT1	ND[a]
GAT2	1.1
GAT3	ND[a]
GAT4	60.0
BEST1	ND[b]

	**Normalized relative expression**[#]
**GABA-A R subunits**	
α1	2.3
α2	2.8
α3	4.8
α4	57.4
α5	9.5
α6	NA[c]
β1	39.5
β2	4.7
β3	12.9
γ1	4.4
γ2	1.0
γ3	16.4
δ	0.07
ε	1.4
θ	ND[c]
π	ND[c]
ρ1	16.4
ρ2	3.3
ρ3	ND[c]

	**Normalized relative expression**[#]
**CCCs**	
NKCC1	1.2
NKCC2	5.7
KCC1	1.2
KCC2	0.6
KCC3	0.4
KCC4	0.9
NCC	4.9

	**Normalized relative expression**[#]
**VDCCs**	
Ca_V 1.1	4.7
Ca_V 1.2	8.1
Ca_V 1.3	0.5
Ca_V 1.4	4.5
Ca_V 2.1	1.0
Ca_V 2.2	1.9
Ca_V 2.3	53.0
Ca_V 3.1	4.3
Ca_V 3.2	3.5
Ca_V 3.3	2.1

# - Relative expression is determined as indicated in Materials and Methods and data is represented as mean of relative expression in astrocytes normalized to that in microglia from 5 independent experiments.

ND[a] - Not detected in any of the microglia cell preparations
ND[b] - Not detected in any of the astrocyte cell preparations
ND[c] - Not detected in any of the microglia or astrocyte cell preparations
