# Supplementary material for: Toxoplasma-Induced Hypermigration of Primary Cortical Microglia Implicates GABAergic Signaling
Source: Front Cell Infect Microbiol. 2019 Mar 20;9:73. doi: 10.3389/fcimb.2019.00073 (PMC6436526; doi:10.3389/fcimb.2019.00073)
Supplement: Supplementary file 4 [file Table_4.pdf]

Table S4 (in reference to Figure 4). Modulated transcriptional expression of GABAergic and VDCC signaling components in Toxoplasma-challenged primary microglia.

	2 h	4 h	12 h	24 h
**Enzymes**				
GAD65	102.1	2.3	-57.2	-32.2
GAD67	314.6	40.3	11.1	33.2
GABA-T	-27.4	-9.3	-68.0	-38.1

	2 h	4 h	12 h	24 h
**Transporters**				
GAT2	26.4	8.5	-46.9	27.6
GAT4	42.4	-3.3	743.0	478.3
BEST1	187.1	38.8	-63.3	-100.0

	4 h	12 h	24 h
**GABA-A R subunits**			
α1	5.3	-31.9	-28.2
α2	10.0	-66.3	-18.5
α3	59.8	-52.8	-27.0
α4	6.8	76.0	-29.0
α5	35.9	-53.6	4.7
β1	-66.4	-100.0	-100.0
β2	20.0	-45.6	32.3
β3	-1.6	-63.8	-48.9
γ1	-2.6	-75.4	-51.9
γ2	-1.3	-56.5	-24.9
γ3	17.0	-73.3	8.1
δ	-43.7	-58.5	-44.8
ε	-14.1	0.0	-62.8
ρ1	-64.7	-78.0	-100.0
ρ2	84.1	85.0	90.7

	2 h	4 h	12 h	24 h
**CCCs**				
NKCC1	47.1	-18.4	-15.4	61.3
NKCC2	31.2	55.8	-55.0	94.0
KCC1	88.7	77.3	67.4	43.8
KCC2	177.7	85.8	7.4	20.4

KCC3	-21.0	-3.4	-51.7	-30.9
KCC4	27.1	-6.5	-42.3	15.8
NCC	76.2	17.3	-64.3	-46.7

	**4 h**	**24 h**
**VDCCs**		
Ca_V 1.1	18.0	-13.6
Ca_V 1.2	9.8	-28.0
Ca_V 1.3	-12.7	84.2
Ca_V 1.4	157.3	-29.4
Ca_V 2.1	-57.6	-39.0
Ca_V 2.2	8.4	-6.3
Ca_V 2.3	26.2	19.7
Ca_V 3.1	-100.0	-100.0
Ca_V 3.2	11.6	46.9
Ca_V 3.3	32.6	14.0

The relative mRNA expression is determined as detailed in Materials and Methods. Data is represented as mean of percentage increase or decrease of mRNA levels in Toxoplasma-challenged primary microglia in comparison to unchallenged microglia at indicated time point from 2-4 independent experiments.
