# Supplementary material for: Toxoplasma-Induced Hypermigration of Primary Cortical Microglia Implicates GABAergic Signaling
Source: Front Cell Infect Microbiol. 2019 Mar 20;9:73. doi: 10.3389/fcimb.2019.00073 (PMC6436526; doi:10.3389/fcimb.2019.00073)
Supplement: Supplementary file 5 [file Image_1.pdf]

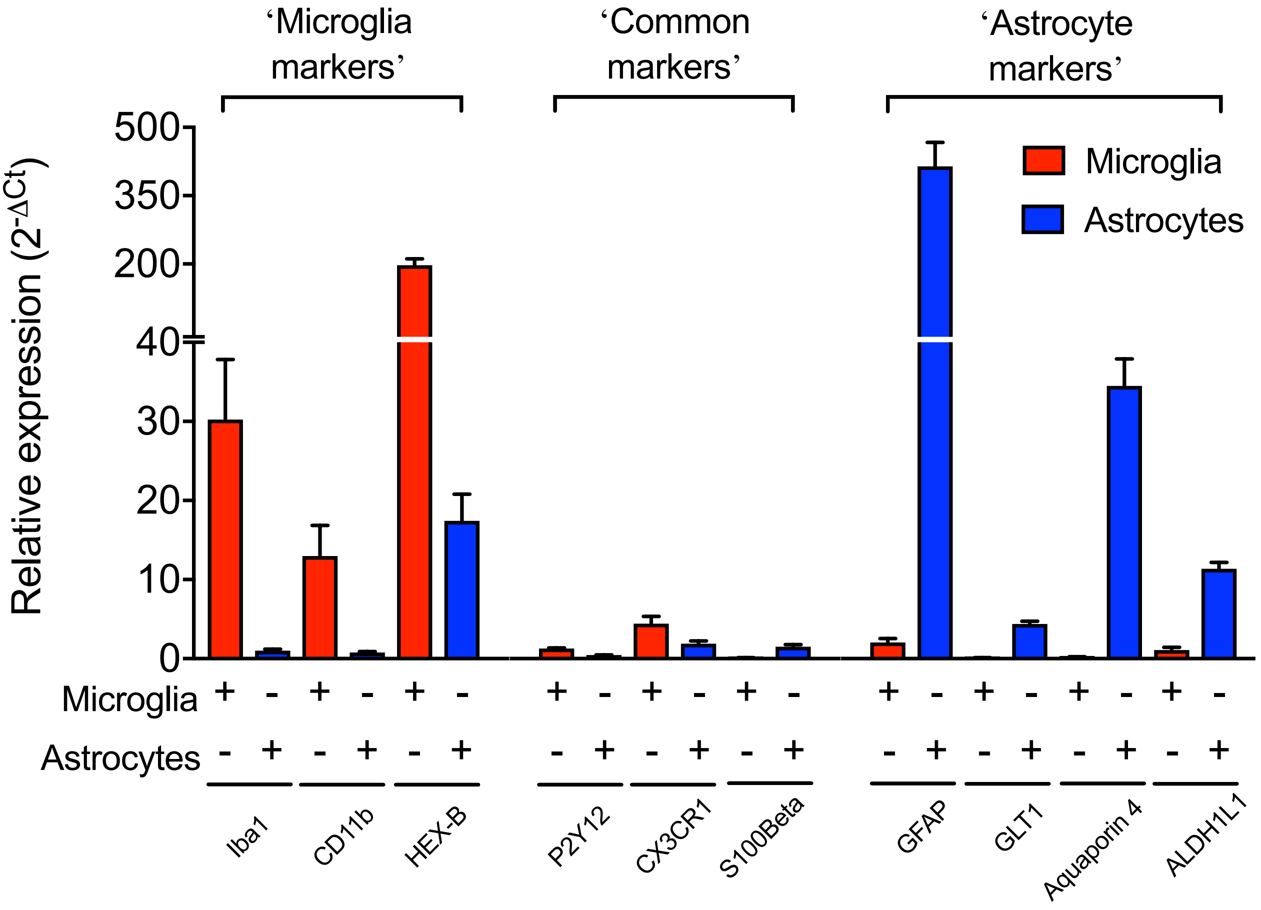

Figure S1. Relative transcriptional expression of microglia and astrocyte markers in microglia and astrocyte cell preparations

The relative mRNA expression ($2^{-\Delta Ct}$) of microglia markers, common markers and astrocyte markers was assessed as indicated under Materials and Methods. Bar graphs represent mean + SEM of 5 independent experiments.
