# Supplementary material for: Toxoplasma-Induced Hypermigration of Primary Cortical Microglia Implicates GABAergic Signaling
Source: Front Cell Infect Microbiol. 2019 Mar 20;9:73. doi: 10.3389/fcimb.2019.00073 (PMC6436526; doi:10.3389/fcimb.2019.00073)
Supplement: Supplementary file 6 [file Image_2.pdf]

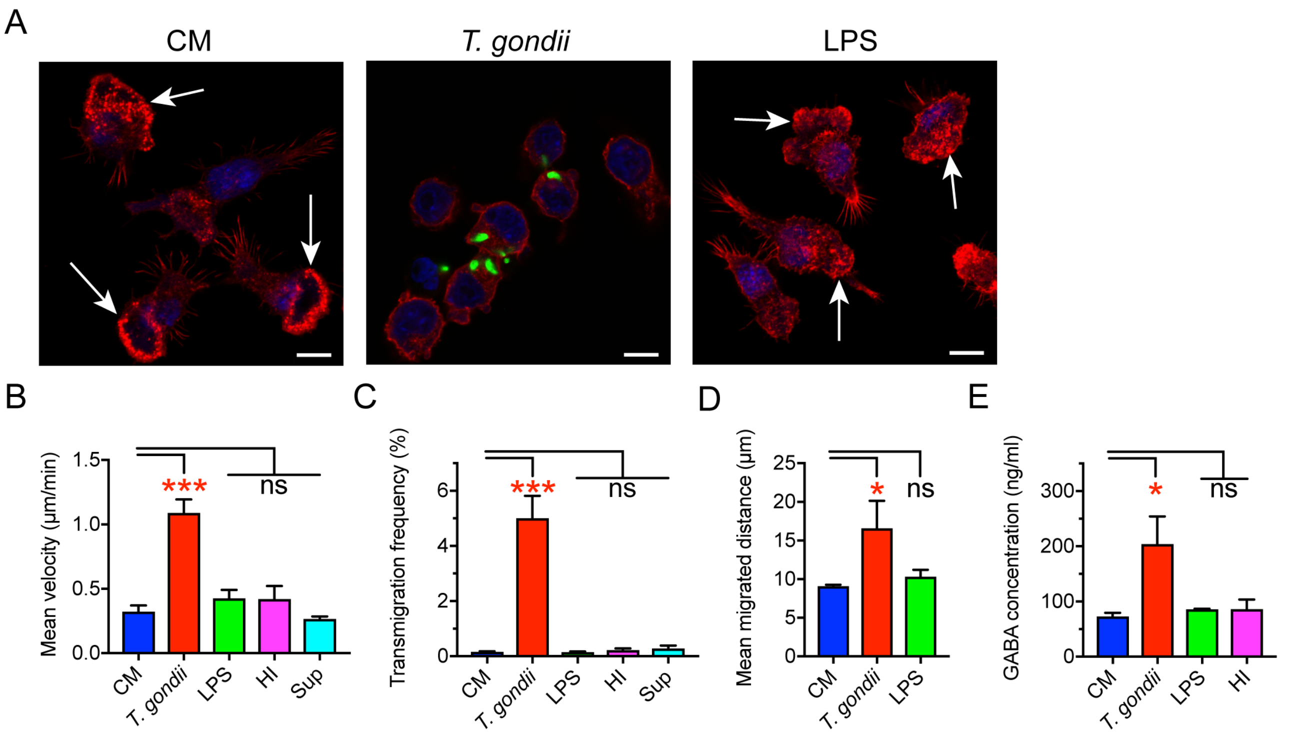

Figure S2. Phenotypic characterization of the microglia cell line BV2 upon challenge with *T. gondii*

(A) Representative micrographs of BV2 cells stained with Alexa Fluor 594 Phalloidin to detect F-actin as indicated under Materials and Methods. Arrows indicate F-actin condensation in podosome structures. Scale bar = 10 µm. **(B)** Mean velocities of BV2 cells incubated for 4 h with complete medium (CM), freshly egressed *T. gondii* tachyzoites (MOI 3), LPS (100 ng/ml), heat-inactivated tachyzoites (HI) or supernatant from *T. gondii*-challenged BV2 cells (Sup) as indicated under Materials and Methods. **(C)** Transmigration frequencies (%) of BV2 cells as in **(B)** incubated for 6 h as indicated under Materials and Methods. **(D)** Mean migrated distances in a 3D matrix by BV2 cells incubated in CM, *T. gondii* tachyzoites or LPS (100 ng/ml) as indicated under Materials and Methods, analyzed from 500 randomly chosen cells.

(E) GABA concentrations in the supernatant of BV2 cells incubated with CM, *T. gondii* tachyzoites, LPS (100 ng/ml) and heat-inactivated tachyzoites (HI). For **(B)**, **(C)**, **(D)** and **(E)**, bar graphs represent mean + SEM of 2-5 independent experiments. For **(B)**, **(C)** and **(E)**, statistical significance was tested by One-Way ANOVA with Dunnett's post hoc test and for **(D)** by Two-way ANOVA with Tukey`s HSD test. ns $p \geq 0.05$, * $p < 0.05$, *** $p < 0.001$.
