# Supplementary material for: Toxoplasma-Induced Hypermigration of Primary Cortical Microglia Implicates GABAergic Signaling
Source: Front Cell Infect Microbiol. 2019 Mar 20;9:73. doi: 10.3389/fcimb.2019.00073 (PMC6436526; doi:10.3389/fcimb.2019.00073)
Supplement: Supplementary file 7 [file Image_3.pdf]

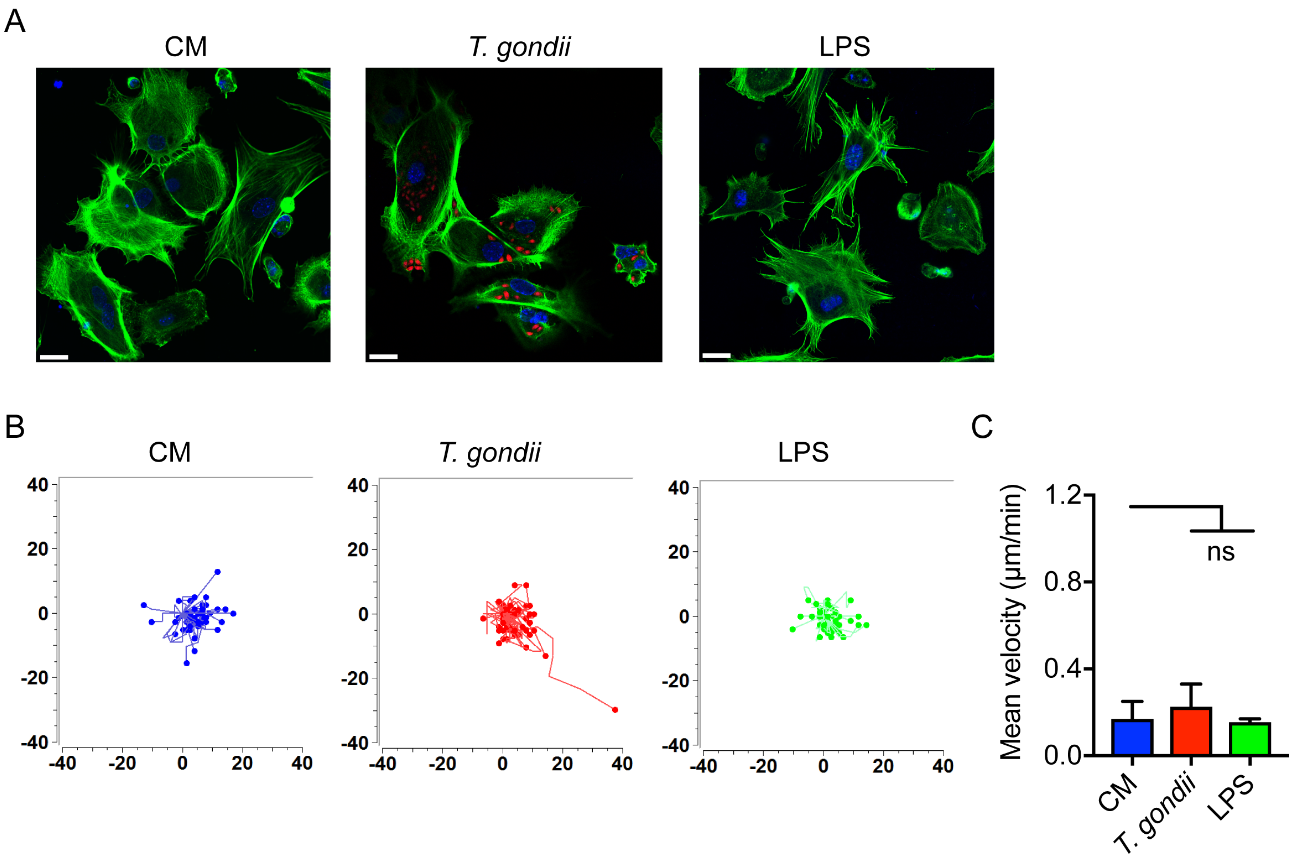

Figure S3. Phenotypic characterization of astrocytes upon challenge with *T. gondii*
(A) Representative micrographs of astrocytes stained with Alexa Fluor Phalloidin to detect F-actin as indicated under Materials and Methods. Scale bar = 10 µm. **(B)** Representative motility plots of astrocytes incubated with complete medium (CM), *T. gondii* tachyzoites or LPS (100 ng/ml) as indicated under Materials and Methods. **(C)** Motility analyses of astrocytes treated as in **(B)**. Bar graphs represent mean velocities + SEM from 2 independent experiments. Statistical significance is tested by One-Way ANOVA with Dunnett's post hoc test. ns $p \geq 0.05$.
